# Acceptability of a proposed new tuberculosis vaccine among people deprived of liberty in Brazil

**DOI:** 10.1016/j.vaccine.2026.128492

**Published:** 2026-03-18

**Authors:** José Victor Bortolotto Bampi, Ghislaine Gonçalez de Araujo Arcanjo, Karina Marques Santos, Michele Souza Ventura, Everton Ferreira Lemos, Rebecca A. Clark, Katherine A. Thomas, Richard G. White, Julio Croda

**Affiliations:** a School of Medicine, Federal University of Mato Grosso do Sul, Av. Costa e Silva, s/n, Bloco 9, Campo Grande, Mato Grosso, do Sul, Brazil; b Oswaldo Cruz Foundation, Rua Gabriel Abrão, 92 - Jardim das Nacoes, Campo Grande, Mato Grosso, do Sul, Brazil; c State University of Mato Grosso do Sul, Av. Dom Antonio Barbosa, 4155, Campo Grande, Mato Grosso, do Sul, Brazil; d Tuberculosis Centre, London, School of Hygiene and Tropical Medicine, Keppel St, WC1E 7HT, London, United Kingdom; e Vaccine Centre, London School of Hygiene and Tropical Medicine, Keppel St, WC1E 7HT, London, United Kingdom; f Department of Epidemiology of Microbial Diseases, Yale University School of Public Health, 60 College St, New Haven, CT, USA

**Keywords:** Tuberculosis vaccine, Vaccine hesitancy, Persons deprived of liberty, Tuberculosis

## Abstract

**Background:**

Tuberculosis (TB) persists as a global threat, with unequal burden across populations. New TB vaccines are in development, and people deprived of liberty (PDL) represent a vulnerable group that may benefit from vaccination. This study evaluated acceptability of a hypothetical new TB vaccine and factors associated with hesitancy among PDL in Brazil.

**Methods:**

We performed a cross-sectional study among PDL in six male and two female prison units from April 2025 to October 2025. From each unit,130 PDL were randomized to be evaluated through a structured questionnaire about sociodemographic status, TB knowledge, acceptability of a hypothetical vaccine and attitudes towards TB vaccination, including Likert-scale statements. We compared characteristics and vaccine attitudes by prison type and by vaccine acceptance status.

**Results:**

Of the 1040 individuals randomized, 945 provided consent to study procedures and were interviewed. Four were excluded due to missing questionnaire answers, with 941 included for main analysis. In total, 95.2% of individuals reported that they would take the TB vaccine if available for them, with 94.1% and 98.7% of acceptance in male and female prisons, respectively. Compared to females, male individuals reported more distrust in vaccine safety (28.9% vs 14.5%, *p* < 0.001), more community coercion to vaccine uptake (15.4% vs 5.6%, p < 0.001) and worse TB knowledge (44.6% vs 31.2%, p < 0.001). Overall, among individuals that would not accept vaccination, 77.8% and 55.6% of them did not trust vaccine safety and efficacy, respectively, 60.0% did not trust healthcare workers and 20.0% reported community coercion.

**Conclusion:**

We found that acceptability of a new TB vaccine in prisons was high. Despite differences in intent to vaccinate regarding gender, individuals that refused vaccination more often reported distrust in vaccine products and healthcare. A significant proportion of them reported community coercion for vaccine uptake. Our findings suggest that a new TB vaccine would be accepted among PDL.

## 1 Introduction

Globally tuberculosis (TB) persists as the leading cause of death from a single infectious agent, affecting over 10 million people in 2025 [1]. Within the End TB Strategy goals there are many proposed approaches to face this global health threat [2], among them, new vaccines that can provide protection against *Mycobacterium tuberculosis* and the development of TB disease in adolescents and adults would be major scientific breakthroughs for disease control across all populational groups [3].

Despite that, there is no current vaccine product licensed for TB protection in adults and adolescents but there are over a dozen candidates in the development pipeline using different platforms [4], many of those already in phase 2 and 3 clinical studies as the most recent recombinant protein vaccine M72/AS01_E–4_ the live-attenuated MTBVAC candidates [5,6]. Within this thriving reality of possibilities for interventions trials and future immunization campaigns, it is paramount that we explore vaccine acceptability, hesitancy and overall perception of these circumstances amid the targeted populations.

Since 2000, the population of people deprived of liberty (PDL) (i.e. individuals detained in remand or convicted and sentenced) has increased 26.9% globally, however, in Brazil, the PDL population has more than tripled in the same period [7]. Currently, TB incidence rates in prison settings are among the highest recorded around the globe [8]; despite constituting less than 1% of the national population, 9.1% of the incident cases of TB in Brazil occur in PDL, amounting to an incidence of 906.4 cases per 100.000 people in 2023 [9]. Within this growing population, overcrowding of Brazilian prisons has reached 140.5% in 2025 [10], with densely populated and poorly ventilated cells that can sustain disease transmission chains [11]; time of exposure to this environment has also been associated to TB in previous studies [12,13].

Current evidence suggest that this high burden population act as reservoirs of disease, with spillover to the overall community [14,15]. In Latin America, previous studies have shown that the increasing incarceration rate in the last decade is one of the main factors for the increased TB incidence overall, amounting to TB rates 26 times higher in prisons compared with the national averages [16,17]. Targeted vaccination campaigns for PDL could add substantially to TB control nationally, thus, it is essential that such a vulnerable population is assessed for willingness to participate in interventions that could reduce the burden of disease.

Most available studies evaluating vaccine acceptancy in prisons are related to high income countries or COVID-19 vaccines [18–20]. The reported coverage and acceptance are variable; however, barriers for coverage as distrust in vaccine products safety and efficacy, distrust in prison staff members and healthcare workers are commonly reported in those studies. In Brazil, despite recent decreases in coverage rates of Measles, Diphtheria-tetanus-pertussis and Poliomyelitis vaccines, vaccination has historical high acceptance in the overall population [21,22]. Yet evidence regarding PDL is scarce; reports from government agencies [23] and individuals states [24] indicate that acceptability of COVID-19 vaccines was high within prisons, however there is no official country level data.

Nevertheless, factors leading to vaccine uptake are complex and have variable determinants amidst populations, vaccine products and targeted diseases [22,25]; meaning that current data might not reflect acceptability for a new TB product. Elements such as TB stigma within prisons, individual risk perception, trust in healthcare workers and information sources [26–28] might unpredictably influence immunization mobilizations success. There are frequent vaccination campaigns performed in Brazilian prisons and currently no law requires PDL participate in these campaigns without their consent, however, previous studies have indicated that PDL might feel coercion by prison staff or other incarcerated individuals to accept these interventions [26].

On account of these knowledge gaps, this study aims to evaluate acceptability and hesitation to a new hypothetical TB vaccine, as well as TB beliefs and knowledge, through a survey performed in 6 male and 2 female prisons across 6 cities in Brazil.

## 2 Material and methods

### 2.1 Study setting and design

We conducted a cross-sectional study at six male prison units located within six cities (Belo Horizonte, Campo Grande, Manaus, Porto Velho, Salvador and Venâncio Aires) and two female prison units located within two cities (Campo Grande and Manaus) in Brazil, covering all country regions. Data collection and study procedures occurred between April 2025 and October 2025.

All units are adult (over 18 years old) medium security prisons, operating in a closed regimen where individuals cannot leave prison during incarceration. In the most recent Brazilian justice department report of 2025, total male prison population varied between 606 (Venâncio Aires) and 1593 (Campo Grande) PDL, the mean occupancy rate was 158%, varying between 105% (Salvador) and 360% (Campo Grande). The female prisons had a population of 220 individuals with 111% of occupancy (Manaus) and 311 individuals with 137% occupancy (Campo Grande) [29].

Only two units, Campo Grande male and female prisons, routinely perform screening for TB at prison entry, the remaining units rely on passive diagnosis alone. Brazilian national health authority does not report incidence rates for individual prison units, however previous studies in Campo Grande have shown an active TB prevalence of over 4000 cases per 100.000 persons in the male unit in 2021 [30] and 746 cases per 100.000 persons in the female unit [13] in 2013. According to Brazilian guidelines, none of the units perform isolation for active TB cases [31].

### 2.2 Data collection

We obtained a complete prison unit census from each unit in the week prior to study procedures and performed a random selection of potential participants based on a prison identification number. After randomization, we obtained the name of the participants and their cell block and cell number to be approached by study personnel. Since study procedures were performed inside the cell blocks, prison staff required to know the individuals we would approach to open cell blocks and provide access for the study personnel, however prison staff were not present during the data collection either was informed about willingness of the individuals to participate. There were no incentives, monetary or otherwise, for individuals to partake in study procedures.

Participant selection was performed to maximize the chance of a sample of 120 PDL for each unit, so we randomized 130 individuals in each prison to account for losses and refusals. We excluded individuals that did not consent to participate and illiterate individuals that would not be able to read the informed consent form. The initial sampled individuals were interviewed by study personnel and inquired about willingness to participate in study procedures. Due to security reasons that were determined by prison staff in each prison, we could only collect data from a limited number of patients during a limited number of days, so recruitment stopped when the sample available was closest to 120 participants for each unit.

**Fig. 1:**
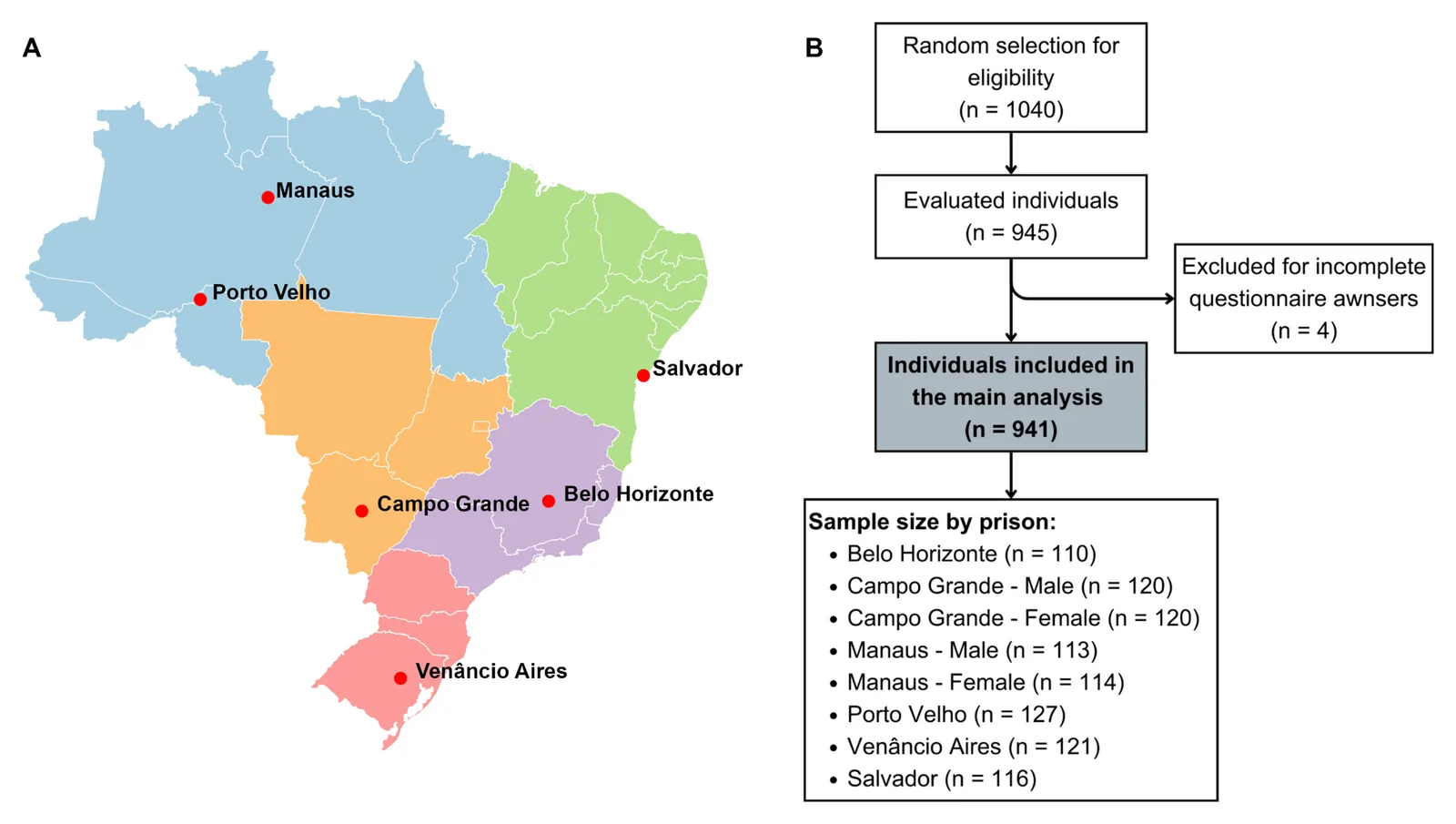
Cities included in the study evaluation and flowchart of inclusion of study participants. **A –** Cities where the study prison units were located within Brazil. Five different country regions are represented in different colors, at least one prison was evaluated in each region. **B –** Flowchart of participant inclusion and number of participants at each site.

All of those who were willing and provided informed consent were interviewed by a trained study researcher that collected answers to questions in a structured sociodemographic and vaccine acceptability questionnaire in paper, that was later transferred to electronic format. After data collection and sample processing, we excluded individuals with incomplete questionnaire answers recorded from the main analysis (Fig. 1).

### 2.3 Vaccine acceptability questionnaire

Participants were inquired about vaccine acceptability through a standardized questionnaire, available in the annex in the original Portuguese version and English translation (Supplementary materials, Appendix 1). The questions were posed in the present tense. Questions related to healthcare workers trust were not structured to assess trust in the prison system workers, but healthcare professionals in general.

Initially, study investigators questioned directly about prior TB knowledge and, utilizing the Likert scale (“Strongly agree”, “Agree”, “Don't know”, “Disagree” and “Strongly disagree”), requested participants to assess affirmations about TB disease concerns.

Currently, since there are no licensed TB vaccines in use for the adult population, we made clear that there is no current adolescent/adult TB vaccine available and study investigators solicited that participants would think about a scenario were a new vaccine was available for them, affirming: “If a new vaccine for adolescents/adults was approved for tuberculosis (TB) and recommended by the Brazil Ministry of Health, how strongly do you agree or disagree with the following affirmations?”. This statement was followed up by affirmations assessing vaccine trust and healthcare trust. After that, individuals were questioned directly about the decision to take the possible vaccine, “If a new vaccine for adolescents/adults was approved for tuberculosis (TB), available to you today, recommended by the Brazil Ministry of Health, and your healthcare provider recommended it, would you choose to get the vaccine?”. Interviewed individuals could choose between the answers “Yes”, “No” and “Unsure”; for analysis purposes we considered acceptance as those who answered “Yes” and aggregated the “No / Unsure” answers as hesitant. Finally, individuals were asked to select options from a multiple-choice list of what they considered trustworthy sources of information for TB vaccine recommendations and other reasons they might consider not taking a TB vaccine if it were available.

### 2.4 Indicators of possible vaccine hesitancy

We decided to utilize the 5C vaccine hesitancy model [32] to approach possible drivers of vaccine denial in future realistic vaccination campaigns scenarios. Through a composite analysis of answers provided, we assessed six categories of possible obstacles to vaccine uptake within the 5C model.

**Confidence** was evaluated through distrust in vaccine (efficacy and safety) and healthcare system. Composite distrust in vaccine safety was considered present if individuals answered “Disagree” or “Strongly disagree” to “I think a new tuberculosis (TB) vaccine would be safe” or if they reported “Health-related barriers”, “Pregnancy or lactating barriers”, “Distrust in vaccines in general” or “Distrust in TB vaccines” among reasons to not take the vaccine. Composite distrust in vaccine efficacy was considered present if individuals answered “Disagree” or “Strongly disagree” to “I think a new tuberculosis (TB) vaccine would be effective” or if they reported “Distrust in TB vaccines” among reasons to not take the vaccine. Composite distrust in healthcare providers and workers was considered present if individuals answered “Disagree” or “Strongly disagree” to “ I would trust the health workers who would give me a new tuberculosis (TB) vaccine”, if they not selected “Facility-based healthcare workers or community/lay healthcare worker (health care professionals)” or “Ministry of Health or other government officials (government agencies)” as trustworthy sources of information or if they selected “I do not trust the healthcare system and/or the healthcare workers” in respect of reasons to not take the vaccine.

**Complacency** was assessed as concerns about TB disease, we considered it present if individuals answered “Agree” OR “Strongly Agree” to the affirmations “I am concerned that I could develop TB” or “If I developed TB, I am concerned that I would become seriously ill or die” and did not select the option “TB beliefs (e.g., I am not concerned about developing TB)” in the reasons they might not take the vaccine.

**Calculation** was evaluated as insufficient TB knowledge if individuals did not report previous TB treatment or if they answered “I had heard of TB and did not knew much about it” or “I have never heard of TB before” to the question “Before today, how much did you know about tuberculosis (TB)?”. We also considered as not adequate information sources if individuals did not select “Facility-based healthcare workers or community/lay healthcare worker (health care professionals)” or “Ministry of Health or other government officials (government agencies)” as trustworthy sources of information.

Although we did not questioned individuals directly about Constrains of access to the vaccine or Collective responsibility about community transmission, we considered a composite variable of Collective constrains, where these individuals indicated that community factors would influence their informed decisions to take or not the vaccine. This was considered present if participants selected “Family or community-related barriers”, “Fear of repression from other people deprived of liberty” or “I don’t believe that i would have the option to deny a TB vaccine, even if i did not wanted to take it” in the reasons they might not take the vaccine.

### 2.5 Data management and statistical analysis

All data collected was stored in an online RedCap® (Vanderbilt University, Tennessee, USA) database hosted by Fiocruz, Mato Grosso do Sul, Brazil. Data analysis was performed utilizing R statistical software [33]. We utilized Chi-square and Fisher tests for categorical variables to determine differences in the response variables among subgroups.

## 3 Results

During the study period, 945 individuals were evaluated through study procedures from 1040 PDL selected previously. Initially, we excluded 4 individuals with incomplete questionnaires, amounting to a final sample of 941 individuals from the 8 prisons included in the main analysis **(**Fig. 1**)**. Overall, 75.1% of PDL were from male prisons, the median age was 32 years old (IQR 27–39) and most were mixed-race (58.0%). More frequently, individuals were single (59.4%) had incomplete basic education (48.4%), had a total family income up to 2 minimum wages (62.2%) and more than half reported receiving visitors during incarceration (56.3%). Current incarceration time was less than a year for around one third (37.9%) of participants and most (71.9%) had a history of previous incarceration. We encountered a high prevalence of self-reported alcohol use disorder (4.5%), illicit drug use (15.3%) and current tobacco use (25.8%), however reported HIV positive status was low (1.4%), similar to the overall Brazilian population estimates [34]. The proportion of previous TB treatment reported was considerable at 9.7% and 34.3% reported previous contact with a positive TB individual in the same cell. Male and female prisons had substantial differences in the previous reported characteristics, but the proportion of HIV positive status, previous TB history and family income did not statistically differ between the groups **(**Table 1**)**.

Most of the participants interviewed (95.2%) in all prisons reported that they would take the new vaccine if available to them. Among the 45 participants that said they would not accept it, only three (6.6%, 3/45) were from female prisons. Participants that would decline a new TB vaccine were very similar to those who would accept a vaccine regarding sociodemographic and prison characteristics. However, we identified that those that would decline a vaccine more frequently reported tobacco smoking (44.4% vs 24.9% *p* = 0.003) and current drug use (26.7% vs. 14.7%, *p* = 0.030). (Supplementary Table 1).

**Fig. 2:**
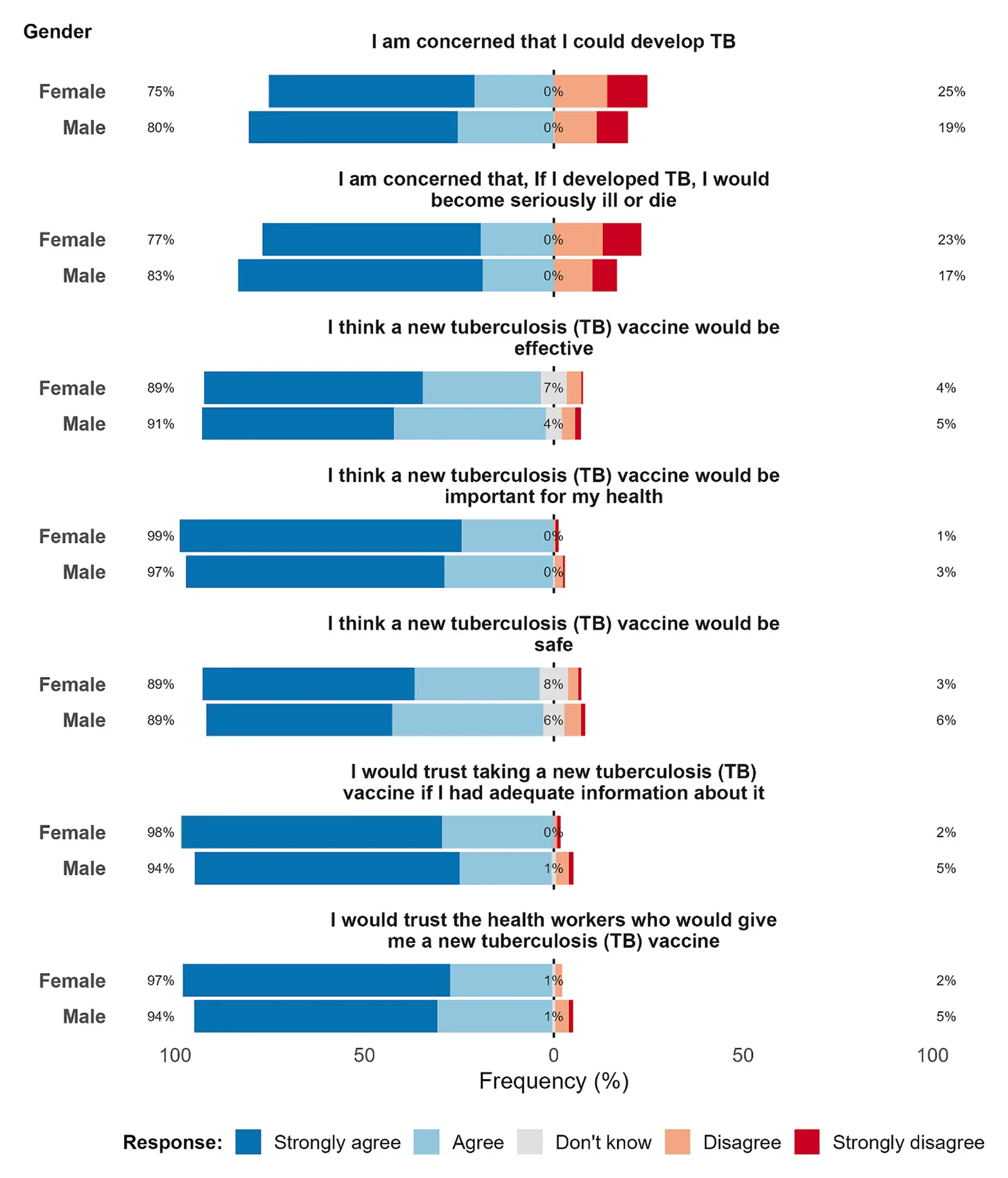
Likert scale responses to questions related to tuberculosis concerns and vaccine acceptability among people deprived of liberty, stratified by willingness to take a proposed TB vaccine (*N* = 941). The Likert awnsers were scaled to center “Don't know” awnsers. The percentages on the left side represent the combined agreement and on the right side the combined disagreement. The values in the middle represent the percentage of “Don't know” awnsers. Results are stratified by hypotetical vaccine acceptance.

Participants that would accept a vaccination had very low disagreement rates with answers related to confidence in the Likert scale answers. (Fig. 2). We also identified that trust in healthcare workers (64.4% vs 81.6%, *p* = 0.004), the Brazilian government (42.2% vs 58.8%, *p* = 0.028) and community leaders (0% vs 8.0%, *p* = 0.042) was lower in those who refused the vaccine. Those individuals refusing the hypothetical vaccine also reported more causes related to health barriers (31.1% vs. 13.5%, *p* < 0.001) to not accept it, distrust was also significantly more frequently reported by the same group, more importantly regarding distrust in general vaccines (28.9% vs. 5.8% *p* < 0.001), TB vaccines (37.8% vs 5.0%, p < 0.001) and healthcare workers (8.9% vs 1.2%, p = 0.004) **(**Table 2**)**.

Despite the small proportion of participants declining a new TB vaccine, we performed the 5C composite analysis comparing the groups of those who would accept vaccination with the refusals and unsure individuals. Those who would not take the vaccine had more distrust in vaccine safety (77.8% vs 22.7%, p < 0.001), efficacy (55.6% vs 7.8%, p < 0.001) and healthcare (60.0% vs 7.9%, p < 0.001) than those who would accept vaccination. These individuals declining vaccination also more frequently reported not having adequate sources of information, not considering healthcare workers or government agencies as reliable (24.4% vs 5.5%, p < 0.001). Although both groups had high proportions of people concerned about TB disease, those who would decline the vaccine were less concerned about TB infection or disease severity, as evaluated by the complacency composite variable (77.8% vs 88.8%, *p* = 0.024). There was no difference in the composite evalation for community coercion between the groups, assessing the answers to family or other PDLs influencing vaccine decision, however, it was reported by a significant proportion of the overall population (13.0%) **(**Table 3**)**.

There were also gender related differences across the responses. Males reported that more “had heard of TB, but did not know much about it” or “had never heard of TB” more than females (44.6% vs 31.2%). We did not find a significant difference in knowledge between prisons that perform TB screening (Campo Grande male and female units) and those who don’t (39.8% vs 41.6%).Among the reasons to deny vaccination, male PDL reported more health-related (18.1% vs 3.0%, *p* < 0.001) and community related barriers (8.1% vs 3.0%, *p* = 0.008) than females. Compared to females, male PDL trusted the facility-based healthcare more (82.7% vs 74.8%, *p* = 0.007) but there was no significant difference in confidence regarding other sources of information (Supplementary Table 2). Responses to the Likert scale questions evidenced that females disagree more about statements regarding their concerns of TB disease (19% vs 25%) and severity (23% vs 17%) than males, however overall agreement with vaccine importance was high and similar between males and females (Supplementary Fig. 1). The 5C composite analysis showed that male PDL have more distrust in vaccine safety (28.9% vs 14.5%, p < 0.001), more community coercion regarding vaccine decisions (15.4% vs 5.6%, p < 0.001) and reported more insufficient previous TB knowledge (44.6% vs 31.3% p < 0.001) compared to females (Supplementary Table 3), despite male prisons presenting with a higher prevalence of previous TB and proportion of people reporting contact with TB cases in the same cell.

## 4 Discussion

In this cross-sectional evaluation of six male and two female prisons in Brazil we detected a very high reported acceptance rate for a proposed new TB vaccine. Participants reporting that they would decline a potential new TB vaccine had a higher proportion of reported active smoking and illicit drug use; the main reasons reported for declining it were related to confidence in the vaccine efficacy and safety, as well as distrust of healthcare professionals and government official sources. We also encountered a lower rate of concern about TB disease among those that declined uptake of a new TB vaccine Surprisingly, 22.7% and 7.8% of individuals that accepted vaccination also reported some distrust in vaccine safety and efficacy, respectively. To our knowledge, this is the first study to evaluate acceptance of a possible new TB vaccine in carceral settings.

The high vaccine acceptability in our population of PDL across genders, is compatible with national data for other vaccines. The Brazilian national immunization program has a history of over half a century successfully implementing vaccination campaigns, promoting vaccine propaganda and presenting successful coverage results leading to a scenario of low hesitancy rates in the general population [35,36]. Despite reductions in the overall coverage in the last decade due to the surge of anti-vaccine movements and reduction of public investment in healthcare [37], vaccine hesitancy remained low regardless of the increased distrust movement in the initial distributions of COVID-19 vaccines [38].

As evidenced by our findings, higher vaccine hesitation has been reported previously to be associated with illicit drug use and tobacco smoking [39,40]. This finding is concerning since our sample evidences a high proportion of reported drug use and that one quarter of the individuals are current active tobacco smokers, this pattern of substance use is even higher in carceral settings worldwide [41]. These specific groups can be evaluated in targeted approaches if future vaccine uptake proves to be lower than expected.

We observed elevated rates of confidence issues, even among those who would accept vaccination. Overall, a quarter of PDL have some distrust in vaccine safety and a tenth reported distrust in vaccine efficacy or healthcare system and professionals. Distrust in these factors is a strong determinant of vaccine acceptability [25,42]; previous surveys conducted in the overall Brazilian population reported that confidence issues were the most relevant motives among people who hesitated in vaccination, with 41.4% and 25.5% of those did not believing that vaccines were safe or effective, respectively [21].

Recent studies have analyzed aspects of vaccine distrust in relation to media and online disinformation in Brazil [43–45]. Cellphone and internet access are restricted within closed system prisons in Brazil, but access to those contents can be obtained through contraband. There is no current evidence on access to those contents by PDL in Brazil, however, since a considerable part of our population had been incarcerated for a short period of time, it is plausible to assume that before their incarceration they might have been exposed to the same disinformation processes that occur in the overall population.

We also need to consider that, in PDL there has historically been ethic abuses, neglect and insufficient access to healthcare take place [26,46]. Recently there has been frequent discussions regarding the inclusion ofPDL in vaccine trials [46]. Studies conducted in prison settings might be more efficient due to the high frequency of endpoints of interest for trials (TB infection and disease), shortening study duration and sample size. However, there are ethical concerns regarding safety and willingness to participate, Previous qualitative research performed in some of the prisons included in this study support our findings that new products can be well accepted and present solutions proposed by the PDL interviewed; increasing vaccine education could strengthen consent and ensuring PDL participation in study oversight with community counsels could promote trust within the population [26]. We hypothesize that this community engagement can increase vaccine acceptability when vaccination campaigns start.

Vaccine education efforts should also be aligned with TB disease education. Previous studies with COVID-19 have also suggested that education about disease could improve vaccine uptake in prisons [47,48]. Our findings highlighted that more concerns about TB and previously knowing someone with TB were positively associated with vaccine acceptance. Willingness to participate in TB vaccine trials has been associated with disease knowledge in other contexts [49], finding that could also represent a possible association with vaccine acceptance since PDL report fear of TB disease for personal or second hand experience as factors influencing vaccination choices [26]. In prison settings, the high incidence of TB [8] leads to the high proportion of individuals reporting cellmates with TB and knowing someone with a TB diagnosis in the past; however, this contact does not guarantee a good understanding of the disease and personal risks.

Although only a small proportion of all participants reported feeling community coercion influencing their decision, this perception was substantially more common among those who declined vaccination. Future trials and vaccine campaigns must ensure that there are measures in place to respect individual autonomy of decision [46].

Our study has several limitations. Our primary outcome, TB vaccine acceptability, is concerning a hypothetical product that it is not currently available. Within the vaccine approval pipeline unexpected factors such as considerable side effects, efficacy of the vaccine, inadequate safety information or distrust in the approval process might reduce the expected acceptability significantly. Also, there is no standardized measurements or questionnaires for hypothetical vaccination scenarios [50], most hesitancy studies are regarding existing products or compare groups that already were vaccinated with those who refused. Also, our questions related to healthcare trust were posed to assess trust in healthcare as a broad term, not only carceral healthcare workers; PDL that went through bad experiences with healthcare workers during incarceration that may trust them less than their healthcare provider outside of prison [26].

Finally, despite evaluating several prison units in all regions of Brazil, these results might not reflect acceptability in the overall population or PDL in other countries. The burden of TB in prisons, especially in Brazil, is disproportional which might lead to a higher vaccine acceptance due to increased knowledge and concern about the disease. Also, due to social desirability bias, it is possible that participants may have falsely reported that they would accept a new TB vaccine due to fear of conflict or exclusion within the prison community for not accepting interventions that would be beneficial for the broader prison population.

## 5 Conclusions

In conclusion, we identified that most incarcerated individuals in our sample would accept a proposed new TB vaccine. Younger age, tobacco smoking and illicit drug use were associated with lower acceptability. Individuals that refused vaccination reported more often distrust in vaccine safety, efficacy and healthcare system, were less concerned about TB disease and had more inadequate vaccine information sources. Our findings indicate that a new vaccine would be well accepted overall among PDL and that prospective vaccination projects should aim to increase populational trust if adequate acceptance is not reached.

## Supplementary Material

**Appendix A.Supplementary data**

Supplementary data to this article can be found online at https://doi.org/10.1016/j.vaccine.2026.128492.

Supplementary material

## Figures and Tables

**Table 1:** Characteristics of incarcerated individuals included in the vaccine acceptability study, by gender (*n* = 941).

				Gender			
Variables		Overall *N* = 941^*1*^		Male *N* = 707^*1*^	Female *N* = 234^*1*^		p-value^2^
***Sociodemographic characteristics***							
**Age categories**							**0.046**
18–25 years old		166 (17.6%)		125 (17.7%)	41 (17.5%)		
26–35 years old		421 (44.7%)		326 (46.1%)	95 (40.6%)		
36–45 years old		250 (26.6%)		185 (26.2%)	65 (27.8%)		
Above 45 years old		104 (11.1%)		71 (10.0%)	33 (14.1%)		
**Race**							**<0.001**
White		185 (19.7%)		146 (20.7%)	39 (16.7%)		
Black		192 (20.4%)		164 (23.2%)	28 (12.0%)		
Mixed		546 (58.0%)		389 (55.0%)	157 (67.1%)		
Yellow		10 (1.1%)		2 (0.3%)	8 (3.4%)		
Indigenous		8 (0.9%)		6 (0.8%)	2 (0.9%)		
**Marital Status**							**<0.001**
Divorced		42 (4.5%)		26 (3.7%)	16 (6.8%)		
Married		209 (22.2%)		175 (24.8%)	34 (14.5%)		
Single		559 (59.4%)		410 (58.0%)	149 (63.7%)		
Stable Union		123 (13.1%)		93 (13.2%)	30 (12.8%)		
Widowed		8 (0.9%)		3 (0.4%)	5 (2.1%)		
**Schooling**							**<0.001**
Incomplete basic education		455 (48.4%)		369 (52.2%)	86 (36.8%)		
Complete basic education or higher		486 (51.6%)		338 (47.8%)	148 (63.2%)		
**Family income**							0.800
Less than 1 minimum wage		164 (17.4%)		124 (17.5%)	40 (17.1%)		
1–2 minimum wages		422 (44.8%)		315 (44.6%)	107 (45.7%)		
3–4 minimum wages		74 (7.9%)		58 (8.2%)	16 (6.8%)		
>4 minimum wages		47 (5.0%)		38 (5.4%)	9 (3.8%)		
Not informed		234 (24.9%)		172 (24.3%)	62 (26.5%)		
**Recieves visits**		530 (56.3%)		414 (58.5%)	116 (50.0%)		**0.016**
***Prison characteristics***							
**Previously incarcerated**		677 (71.9%)		542 (76.7%)	135 (57.7%)		**<0.001**
**Previous contact with TB case in the same cell**		323 (34.3%)		260 (36.8%)	63 (26.9%)		**0.006**
**Current incarceration time**							**<0.001**
<1 year		357 (37.9%)		221 (31.3%)	136 (58.1%)		
1–2 years		275 (29.2%)		224 (32.7%)	51 (21.8%)		
3–4 years		127 (13.5%)		108 (15.3%)	19 (8.1%)		
5 years or above		182 (19.3%)		154 (21.8%)	28 (12.0%)		
***Clinical characteristics***							
**Previous TB history**		87 (9.2%)		72 (10.2%)	15 (6.4%)		0.084
**Reported living with HIV**		13 (1.4%)		8 (1.1%)	5 (2.1%)		0.300
**Reported alcohol abuse disorder**		42 (4.5%)		41 (5.8%)	1 (0.4%)		**<0.001**
**Current tobacco smoking**		243 (25.8%)		205 (29.0%)	38 (17.3%)		**<0.001**
**Illicit drug use**		144 (15.3%)		122 (17.3%)	22 (9.4%)		**0.004**

2 Wilcoxon rank sum test; Fisher's exact test; Pearson's Chi-squared test

**Table 2:** Vaccine acceptability questions assessing knowledge, information sources and reasons to not get vaccinated in people deprived of liberty, by willingness to take a proposed TB vaccine (n = 941).

				Would you take the TB vaccine?			
Variables		Overall *N* = 941^1^		No / Unsure *N* = 45^1^	Yes *N* = 896^1^		p-value^2^
**“Before today, how much did you know about tuberculosis (TB)?”**							0.800
I had heard of TB, and knew a lot about it		189 (20.1%)		8 (17.8%)	181 (20.2%)		
I had heard of TB, and knew some things about it		364 (38.7%)		19 (42.2%)	345 (38.5%)		
I had heard of TB, but did not know much about it		342 (36.3%)		15 (33.3%)	327 (36.5%)		
I had never heard of TB		46 (4.9%)		3 (6.7%)	43 (4.8%)		
**“Has someone you know ever had tuberculosis (TB)?”**							**0.049**
Yes, someone I know has had or currently has TB		594 (63.1%)		30 (66.7%)	564 (62.9%)		
No, I do not know anyone who has had TB		329 (35.0%)		12 (26.7%)	317 (35.4%)		
Unsure		18 (1.9%)		3 (6.7%)	15 (1.7%)		
***What sources of information would you trust the most to recommend a new TB vaccine?***							
Facility-based healthcare workers or community/lay healthcare worker (health care professionals)		760 (80.8%)		29 (64.4%)	731 (81.6%)		**0.004**
Family, friends, colleagues (personal network)		369 (39.2%)		13 (28.9%)	356 (39.7%)		0.150
Ministry of Health or other government officials (government agencies)		546 (58.0%)		19 (42.2%)	527 (58.8%)		**0.028**
Religious leaders (faith)		140 (14.9%)		3 (6.7%)	137 (15.3%)		0.110
Other community leaders (community-based network)		72 (7.7%)		0 (0%)	72 (8.0%)		**0.042**
Social media/internet sources		77 (8.2%)		1 (2.2%)	76 (8.5%)		0.200
Other		12 (1.3%)		4 (8.9%)	8 (0.9%)		**0.002**
***What are some reasons you might not get a TB vaccine if it were available to you?***							
Health-related barriers (e.g. allergies or other health conditions)		135 (14.3%)		14 (31.1%)	121 (13.5%)		**0.001**
Pregnancy or lactating barriers (e.g., safety while pregnant or breastfeeding)		12 (1.2%)		1 (3.0%)	11 (1.2%)		0.150
Family or community-related barriers (e.g., fear of reaction/ stigma)		64 (6.8%)		4 (8.9%)	60 (6.7%)		0.500
TB beliefs (e.g., I am not concerned about developing TB)		9 (1.0%)		2 (4.4%)	7 (0.8%)		0.065
Distrust in vaccines in general (e.g., negative experiences with vaccines, I do not like injections, I am concerned about potential side effects of vaccines [e.g., long-term or short-term side effects including fertility])		65 (6.9%)		13 (28.9%)	52 (5.8%)		**<0.001**
Distrust in TB vaccines (e.g., TB vaccine is not important or necessary, I do not think a new TB vaccine will work or stop me from getting TB, I do not think a new TB vaccine will be safe, or I am concerned about potential side effects of a new TB vaccine)		62 (6.6%)		17 (37.8%)	45 (5.0%)		**<0.001**
I do not trust the healthcare system and/or the healthcare workers		15 (1.6%)		4 (8.9%)	11 (1.2%)		**0.004**
Fear of repression from other people deprived of liberty		14 (1.5%)		1 (2.2%)	13 (1.5%)		0.500
I don't believe that i would have the option to deny a TB vaccine, even if I did not wanted to take it		59 (6.3%)		5 (11.1%)	54 (6.0%)		0.200
None of the above		625 (66.4%)		9 (20.0%)	616 (68.8%)		**<0.001**
Other		20 (2.1%)		1 (2.2%)	19 (2.1%)		>0.900

1 n (%)

2 Fisher’s exact test; Pearson’s Chi-squared test

**Table 3:** Variables associated with not accepting a new proposed TB vaccine among people deprived of liberty in Brazil (n = 941).

				Would you take the TB vaccine?			
Variables		Overall N = 941^1^		No / Unsure N = 45^1^	Yes N = 896^1^		p-value^2^
***Confidence***							
Composite distrust in vaccine safety		238 (25.3%)		35 (77.8%)	203 (22.7%)		**<0.001**
Composite distrust in vaccine efficacy		95 (10.1%)		25 (55.6%)	70 (7.8%)		**<0.001**
Composite distrust in healthcare system/workers		98 (10.4%)		27 (60.0%)	71 (7.9%)		**<0.001**
***Complacency***							
Concerned about TB		831 (88.3%)		35 (77.8%)	796 (88.8%)		**0.024**
***Calculation***							
Bad previous TB knowledge		388 (41.2%)		18 (40.0%)	370 (41.3%)		0.900
Not adequate sources of information		60 (6.4%)		11 (24.4%)	49 (5.5%)		**<0.001**
***Collective constrains***							
Reported community coercion		122 (13.0%)		9 (20.0%)	113 (13.0%)		0.150

1 n (%)

2 Pearson's Chi-squared test; Fisher's exact test

## Data Availability

Data will be made available on request.
