# Supplementary material for: Acceptability of a proposed new tuberculosis vaccine among people deprived of liberty in Brazil
Source: Vaccine. Author manuscript; Available in PMC 2026 Oct 30. (PMC7619656; doi:10.1016/j.vaccine.2026.128492)
Supplement: Supplementary file 1 [file EMS220160-supplement-Supplementary_material.docx]

**SUPPLEMENTARY MATERIAL – APPENDIX 1**

**VACCINE ACCEPTABILITY QUESTIONNAIRE**

1. Original Portuguese questionnaire applied in study procedures -------------------------------- Page 2
2. Translated vaccine acceptability questionnaire in English ---------------------------------------- Page 6

**Percepções da população privada de liberdade em relação às pesquisas sobre tuberculose e vacinas**

**1. Identificação**

| Unidade prisional: |  | Pavilhão: |  | Bloco: |  | Cela: |  |
| --- | --- | --- | --- | --- | --- | --- | --- |

| 1.1 Nome: |  | | | | | | | |
| --- | --- | --- | --- | --- | --- | --- | --- | --- |
| 1.2 Nome da mãe: | |  | | | | | | |
| 1.3 Data de nascimento: | | |  | / |  | / |  |  |

1.4 Sexo:

⚪ Masculino

⚪ Feminino

1.5 Raça/cor:

⚪ Branca

⚪ Preta

⚪ Parda

⚪ Amarela

⚪ Indígena

⚪ Sem declaração

| 1.6 RGI (registro do interno): |  | |
| --- | --- | --- |
| 1.7 Cidade/Estado de residência antes da prisão: | |  |

**2. Dados sociodemográficos**

2.1 Estado civil:

⚪ Solteiro/a

⚪ Casado/a

⚪ Viúvo/a

⚪ Divorciado/a

⚪ União estável

2.2 Escolaridade:

| ⚪ Sem escolaridade  ⚪ 1-4 série do ensino fundamental incompleto  ⚪ 1-4 série do ensino fundamental completo  ⚪ 5-9 série do ensino fundamental incompleto  ⚪ 5-9 série do ensino fundamental completo | ⚪ Ensino médio incompleto  ⚪ Ensino médio completo  ⚪ Ensino superior incompleto  ⚪ Ensino superior completo  ⚪ Pós-graduação |
| --- | --- |

2.3 Renda familiar:

⚪ Menos de 1 salário mínimo (< R$ 1.518)

⚪ 1 a 2 salários mínimos (R$ 1.518 a 3.036)

⚪ 3 a 4 salários mínimos (R$ 4.554 a 6.072)

⚪ Mais de 4 salários mínimos (> R$ 6.072)

⚪ Não informado

**3. Comorbidades (problemas de saúde) / fatores de risco**

*pode selecionar mais de uma opção

| ⬜ Desnutrição  ⬜ Diabetes tipo 1  ⬜ Diabetes tipo 2  ⬜ Infecção pelo HIV  ⬜ Tabagismo  ⬜ Transtorno por uso de álcool | ⬜ Uso de substâncias ilícitas  ⬜ Tuberculose (TB)  ⬜ Doença renal  ⬜ Doença hematológica  ⬜ Doença hepática  ⬜ Obesidade | ⬜ Imunodeficiência  ⬜ Doença neurológica  ⬜ Doença pulmonar  ⬜ Asma  ⬜ Outras comorbidades, especificar  ___________________________ |
| --- | --- | --- |

**4. Dados da investigação**

4.1. Tempo que está preso/a:

| ⚪ 1 ano  ⚪ 2 anos  ⚪ 3 anos  ⚪ 4 anos | ⚪ 5 anos  ⚪ 6 a 9 anos  ⚪ 10 ou mais |  |  |
| --- | --- | --- | --- |

4.2 Houve mudança de UP:

⚪Sim, passou por quantas UP:_________________________________________________________________

⚪ Não

4.3. Já esteve preso/a anteriormente:

⚪ Sim, por quanto tempo: _____________________________________________________________________

⚪ Não

4.4 Recebe visita:

⚪ Sim, qual a frequência: _____________________________________________________________________

⚪ Não

Se sim, de (pode selecionar mais de uma opção):

| ⬜ Pai/Padrasto  ⬜ Mãe/Madrasta  ⬜ Esposo/a | ⬜ Namorado/a  ⬜ Irmãos  ⬜ Filhos | ⬜ Amigos  ⬜ Advogado  ⬜Outros ________________________________________ |
| --- | --- | --- |

4.5 Teve contato com paciente TB na mesma cela:

⚪ Sim, se souber, especifique o nome: ___________________________________________________________

⚪ Não

⚪ Ignorado

4.6 Está fazendo tratamento de TB:

⚪ Sim

⚪ Não

4.7 Já fez tratamento anterior de TB:

⚪Sim, no ano: ______________________________________________________________________________

⚪ Não

4.8. Faz uso de medicação de uso contínuo:

⚪Sim, qual? __________________________________________________________________________________

⚪ Não

**Aceitabilidade de vacinas**

**A. TB em geral**

1. Antes de hoje, o quanto você sabia sobre a tuberculose (TB)?

⚪ Eu tinha ouvido falar de tuberculose e sabia muito sobre isso

⚪ Eu tinha ouvido falar de tuberculose e sabia algumas coisas sobre ela

⚪ Eu tinha ouvido falar de TB, mas não sabia muito sobre isso

⚪ Eu nunca tinha ouvido falar de TB

2. Que você saiba, você já foi tratado para tuberculose (TB) no passado?

⚪ Sim, já fui tratado para tuberculose

⚪ Não, nunca fui tratado para a doença da tuberculose

⚪ Prefiro não dizer

3. Alguém que você conhece já teve tuberculose (TB)?

⚪ Sim, alguém que conheço teve ou tem tuberculose

⚪ Não, não conheço ninguém que tenha tido tuberculose

⚪ Não sei

4. Quão fortemente você concorda ou discorda das seguintes afirmações?

|  | Concordo totalmente | Concordo | Discordo | Discordo totalmente | Não sei |
| --- | --- | --- | --- | --- | --- |
| Estou preocupado que eu possa desenvolver tuberculose |  |  |  |  |  |
| Se eu desenvolvesse tuberculose, estou preocupado em ficar gravemente doente ou morrer |  |  |  |  |  |

**B. Vacinas contra a tuberculose**

5. Se uma nova vacina contra a tuberculose (TB) for aprovada e recomendada pelo Ministério da Saúde Brasileiro, quão fortemente você concordaria ou discordaria das seguintes afirmações?

|  | Concordo totalmente | Concordo | Discordo | Discordo totalmente | Não sei |
| --- | --- | --- | --- | --- | --- |
| Acho que uma nova vacina contra tuberculose (TB) seria importante para minha saúde |  |  |  |  |  |
| Acho que uma nova vacina contra tuberculose (TB) seria segura |  |  |  |  |  |
| Acho que uma nova vacina contra tuberculose (TB) seria eficaz |  |  |  |  |  |
| Eu confiaria nos profissionais de saúde que me dariam uma nova vacina contra tuberculose (TB) |  |  |  |  |  |
| Eu confiaria em tomar uma nova vacina contra tuberculose (TB) se tivesse informações adequadas sobre ela |  |  |  |  |  |

6. Se uma nova vacina para adolescentes/adultos fosse aprovada para tuberculose (TB), e estivesse disponível para você hoje, recomendada pelo Ministério da Saúde Brasileiro, e seu médico a recomendasse, você escolheria tomar a vacina?

⚪ Sim (pule para pergunta 8)

⚪ Não (pule para pergunta 8)

⚪ Não sei (responda à pergunta 7)

7. *[Se não tiver certeza para a pergunta 6]* Se uma nova vacina contra tuberculose (TB) estivesse disponível hoje e você tivesse que fazer uma escolha, você escolheria tomar a vacina?

⚪ Sim

⚪ Não

8. Em quais fontes de informação você mais confiaria para recomendar uma nova vacina contra a tuberculose? Por favor, selecione todas as opções aplicáveis. Por favor, não responda até que todas as respostas tenham sido lidas.

⬜ Profissionais de saúde baseados em instalações ou agentes de saúde comunitários/leigos (profissionais de saúde)

⬜ Família, amigos, colegas (rede pessoal)

⬜ Ministério da Saúde ou outros funcionários do governo (agências governamentais)

⬜ Líderes religiosos (fé)

⬜ Outros líderes comunitários (rede baseada na comunidade)

⬜ Fontes de mídia social / internet

⬜Outra(s) _________________________________________________________________________________

9. Quais são algumas das razões pelas quais você pode não receber uma vacina contra a tuberculose se ela estiver disponível para você? Por favor, selecione todas as opções aplicáveis. Por favor, não responda até que todas as respostas tenham sido lidas.

⬜ Barreiras relacionadas à saúde (por exemplo, tenho um histórico anterior de alergias ou outras condições de saúde que podem tornar certas vacinas inseguras)

⬜ Barreiras de gravidez ou lactação (por exemplo, tenho preocupações sobre a segurança de receber uma vacina durante a gravidez ou amamentação)

⬜ Barreiras relacionadas à família ou à comunidade (por exemplo, eu estaria preocupado com a reação ou objeções de meu cônjuge, família e/ou comunidade, minha religião proíbe ou desencoraja o recebimento de vacinas, estigma)

⬜ Crenças de TB (por exemplo, não estou preocupado em desenvolver TB)

⬜ Desconfiança nas vacinas em geral (por exemplo, tive experiências negativas anteriores com vacinas, não gosto de injeções, estou preocupado com os possíveis efeitos colaterais das vacinas [por exemplo, efeitos colaterais de longo ou curto prazo, incluindo fertilidade])

⬜ Desconfiança nas vacinas contra a tuberculose (por exemplo, não acho que uma nova vacina contra a tuberculose seja importante ou necessária, não acho que uma nova vacina contra a tuberculose funcione ou me impeça de contrair tuberculose, não acho que uma nova vacina contra a tuberculose será segura ou estou preocupado com os possíveis efeitos colaterais de uma nova vacina contra a tuberculose [por exemplo, efeitos colaterais de longo ou curto prazo, incluindo fertilidade])

⬜ Não confio no sistema de saúde e/ou nos profissionais de saúde

⬜ Medo de repressão por outros encarcerados

⬜ Não sinto que teria a opção de dizer não para receber uma vacina contra a tuberculose, mesmo que não quisesse tomá-la

⬜ Nenhuma das opções acima

⬜Outra(s) ____________________________________________________________________________________

**Considerações finais**

Observações:

Data da entrevista: _____/_____/_________

Nome, assinatura ou carimbo do entrevistador: ______________________________________________________

**English translation of the original vaccine questionnaire**

**A. TB knowledge overall**

1. Before today, how much did you know about tuberculosis (TB)?

⚪ I had heard of TB, and knew a lot about it

⚪ I had heard of TB, and knew some things about it

⚪ I had heard of TB, but did not know much about it

⚪ I had never heard of TB

2. To your knowledge, have you ever been treated for tuberculosis (TB) disease in the past?

⚪ Yes, I have previously been treated for TB disease

⚪ No, I have never been treated for TB disease

⚪ I would rather not discuss it

3. Has someone you know ever had tuberculosis (TB)?

⚪ Yes, someone I know has had or currently has TB

⚪ No, I do not know anyone who has had TB

⚪ Unsure

4. How strongly do you agree or disagree with the following affirmations?

|  | Strongly agree | Agree | Disagree | Strongly disagree | Don’t know |
| --- | --- | --- | --- | --- | --- |
| I am concerned that I could develop TB |  |  |  |  |  |
| If I developed TB, I am concerned that I would become seriously ill or die |  |  |  |  |  |

**B. TB vaccines**

5. If a new vaccine for adolescents/adults was approved for tuberculosis (TB) and recommended by the Brazil Ministry of Health, how strongly do you agree or disagree with the following affirmations?

|  | Strongly agree | Agree | Disagree | Strongly disagree | Don’t know |
| --- | --- | --- | --- | --- | --- |
| I think a new tuberculosis (TB) vaccine would be important for my health |  |  |  |  |  |
| I think a new tuberculosis (TB) vaccine would be safe |  |  |  |  |  |
| I think a new tuberculosis (TB) vaccine would be effective |  |  |  |  |  |
| I would trust the health workers who would give me a new tuberculosis (TB) vaccine |  |  |  |  |  |
| I would trust taking a new tuberculosis (TB) vaccine if I had adequate information about it |  |  |  |  |  |

6. If a new vaccine for adolescents/adults was approved for tuberculosis (TB), available to you today, recommended by the Brazil Ministry of Health, and your healthcare provider recommended it, would you choose to get the vaccine?

⚪ Yes (go to question 8)

⚪ No (go to question 8)

⚪ Unsure (awnser to question 7)

7. *[If “Unsure” in question 6]* If a new tuberculosis (TB) vaccine was available today, and you had to make a choice, would you choose to get the vaccine?

⚪ Yes

⚪ No

8. What sources of information would you trust the most to recommend a new TB vaccine? Please, select all applicable options. Please, do not answer until all responses have been read.

⬜ Facility-based healthcare workers or community/lay healthcare worker (health care professionals)

⬜ Family, friends, colleagues (personal network)

⬜ Ministry of Health or other government officials (government agencies)

⬜ Religious leaders (faith)

⬜ Other community leaders (community-based network)

⬜ Social media/internet sources

⬜Other _________________________________________________________________________________

9. What are some reasons you might not get a TB vaccine if it were available to you? Please, select all applicable options. Please, do not answer until all responses have been read.

⬜ Health-related barriers (e.g., I have a prior history of allergies or other health conditions that may make certain vaccines unsafe)

⬜ Pregnancy or lactating barriers (e.g., I have concerns about the safety of receiving a vaccine while pregnant or breastfeeding)

⬜ Family or community-related barriers (e.g., I would be concerned about the reaction or objections from my spouse, family and/or the community, my religion prohibits or discourages receiving vaccines, stigma)

⬜ TB beliefs (e.g., I am not concerned about developing TB)

⬜ Distrust in vaccines in general (e.g., I have had previous negative experiences with vaccines, I do not like injections, I am concerned about potential side effects of vaccines [e.g., long-term or short-term side effects including fertility])

⬜ Distrust in TB vaccines (e.g., I do not think a new TB vaccine is important or necessary, I do not think a new TB vaccine will work or stop me from getting TB, I do not think a new TB vaccine will be safe, or I am concerned about potential side effects of a new TB vaccine [e.g., long-term or short-term side effects including fertility])

⬜ I do not trust the healthcare system and/or the healthcare workers

⬜ Fear of repression from other people deprived of liberty

⬜ I don’t believe that I would have the option to deny a TB vaccine, even if i did not wanted to take it

⬜ None of the above

⬜Other ____________________________________________________________________________________
